# Every fifth patient suffered a high nutritional risk—Results of a prospective patient survey in an oncological outpatient center

**DOI:** 10.3389/fnut.2022.1033265

**Published:** 2022-10-31

**Authors:** Julia Jendretzki, Dorothea Henniger, Lisa Schiffmann, Constanze Wolz, Anne Kollikowski, Alexander Meining, Hermann Einsele, Marcela Winkler, Claudia Löffler

**Affiliations:** ^1^Comprehensive Cancer Center, University Hospital Würzburg, Würzburg, Germany; ^2^Klinik Hallerwiese-Cnopf’sche Kinderklinik, Neonatologie und Pädiatrie, Nuremberg, Germany; ^3^Department of Internal Medicine II, University Hospital Würzburg, Würzburg, Germany; ^4^Robert-Bosch-Krankenhaus, Stuttgart, Germany

**Keywords:** nutritional risk screening, malnutrition, nutritional counseling, oncology outpatients, MUST-Score, nutritional medical needs

## Abstract

**Introduction:**

Malnutrition in cancer patients often remains undetected and underestimated in clinical practice despite studies revealing prevalences from 20 to 70%. Therefore, this study aimed to identify patient groups exposed to an increased nutritional risk in a university oncological outpatient center.

**Methods:**

Between May 2017 and January 2018 we screened oncological patients there using the malnutrition universal screening tool (MUST). Qualitative data were collected by a questionnaire to learn about patients’ individual information needs and changes in patients’ diets and stressful personal nutrition restrictions.

**Results:**

We included 311 patients with various cancers. 20.3% (*n* = 63) were found to be at high risk of malnutrition, 16.4% (*n* = 51) at moderate risk despite a mean body mass index (BMI) of 26.5 ± 4.7 kg/m^2^. The average age was 62.7 (± 11.8) with equal gender distribution (52% women, *n* = 162). In 94.8% (*n* = 295) unintended weight loss led to MUST scoring. Patients with gastrointestinal tumors (25%, *n* = 78) and patients >65 years (22%, *n* = 68) were at higher risk. Furthermore, there was a significant association between surgery or chemotherapy within six months before survey and a MUST score ≥2 (OR = 3.6). Taste changes, dysphagia, and appetite loss were also particular risk factors (OR = 2.3–3.2). Young, female and normal-weight patients showed most interest in nutrition in cancer. However, only 38% (*n* = 118) had a nutritional counseling.

**Conclusion:**

This study confirms that using the MUST score is a valid screening procedure to identify outpatients at risk of developing malnutrition. Here one in five was at high risk, but only 1% would have been detected by BMI alone. Therefore, an ongoing screening procedure with meaningful parameters should be urgently implemented into the clinical routine of cancer outpatients as recommended in international guidelines.

## Introduction

According to the German Foundation for Disease-Related Malnutrition, more than 50,000 people in Germany die annually not from their underlying disease but from the consequences of malnutrition, including a large proportion of cancer patients (1) where the prevalence of malnutrition varies from 20 to more than 70% (2–4).

A large study investigating weight loss in cancer patients before diagnosis has shown that up to 80% patients particularly with gastrointestinal tumors experienced severe weight loss. Overall, decreased survival was shown when unintentional weight loss occurred (5).

The causes of common weight loss in cancer patients and the development of malnutrition are multifactorial. Systemic inflammation induces changes in energy balance and metabolism (6, 7). However, decreased nutrient absorption also plays a crucial role. This may be due to obstruction of the gastrointestinal tract and painful ulceration, but also to a general lack of appetite and deterioration of the patient’s physical and psychological well-being (8). Furthermore, antitumor therapy and its side effects (e.g., fatigue, nausea, taste changes and mucositis) may induce or aggravate the malnutrition (9). Due to inadequate energy intake and catabolic metabolism, an improvement of nutritional status and (re)building of body cell mass is usually difficult by oral food intake alone, even under supportive nutritional therapy.

A large proportion of cancer patients already have an inadequate nutritional status at diagnosis. This affects patients’ performance, quality of life and prognosis (7, 10), further the length of hospital stay and the likelihood of complications are tripled (11).

Up to now, the prevalence of malnutrition in Germany has been studied mainly among inpatients. The best known is the German Hospital Malnutrition Study by Pirlich et al. published in 2006, when 27% of patients were diagnosed as malnourished (10). Most recently, a 2020 study by Hauner et al. also recorded a high risk of malnutrition in 20–29% of cancer patients among outpatients, depending on the screening procedure (12).

Despite the lack of an internationally uniform definition of standardized criteria for the detection of malnutrition, its clinical relevance is undisputed. In “Clinical Nutrition in Oncology,” the German Society for Nutritional Medicine requests a systematic and routine screening of all hospitalized patients, repeated at regular intervals (13). However, this is not yet regularly implemented in everyday clinical practice. A screening procedure is also recommended for tumor patients’ diagnosis to stabilize the nutritional status and prevent future problems arising from malnutrition (7, 14). Correspondingly, the European Society for Clinical Nutrition and Metabolism (ESPEN) recommended three screening methods in the 2002 guidelines: The Malnutrition Universal Screening Tool (MUST) for outpatients, the Nutritional Risk Screening (NRS-2002) for inpatients, and the Mini Nutritional Assessment (MNA) for geriatric patients (14).

Consequently, our study aims to determine the objective need for nutritional counseling among cancer patients at the Comprehensive Cancer Center of the University Hospital of Wuerzburg. In addition, we aimed to understand what drives patients, regardless of screening results, and how they are navigating the issue of nutrition to provide them with the best possible advice.

## Materials and methods

### Selection of the patient collective

A total of 311 patients at Wuerzburg University Hospital suffering from a hematological or solid tumor and undergoing outpatient treatment were surveyed. The study period was May 2017 to January 2018. The patients had to be at least 18 years old, otherwise there were no exclusion criteria for participation in this study regarding gender, tumor entity, and stage of therapy or disease.

Written informed consent was obtained from each patient after detailed explanation of the interview procedure, consent form, and data protection. Consent could be withdrawn at any time during and after the survey. The study was approved by the Ethics Committee of the Julius-Maximilian-University Wuerzburg on 25 April 2017 (Nstudy number 88/17) and performed according to the Declaration of Helsinki.

### Data collection

First, general patient data as well as data on the stage of the disease and the clinical course of therapy were collected by a MD student. The nutritional status was assessed using the Malnutrition Universal Screening Tool (MUST) screening questionnaire. The second questionnaire obtained subjective assessments from patients regarding their individual need for information on nutrition in cancer, their nutritional status, and current nutritional problems. The interviews only took place at on one day and the data was collected paper based.

### General patient data

The gender and age of each patient were recorded. Furthermore, height and weight were asked to calculate the Body Mass Index (BMI) as well as weight loss in the last six months.

The oncological diagnosis and therapy status were collected, including if and when surgery, chemotherapy or radiotherapy had taken place. The palliative or curative intention of therapy at the time of the survey were noted.

### Malnutrition universal screening tool questionnaire and patient questionnaire

To objectively screen for nutritional risk, we used the validated MUST for outpatient setting, developed by the British Association for Parenteral and Enteral Nutrition (BAPEN) (15). Since 2002 it has been recommended by the ESPEN guidelines and is also part of the DGEM guideline “Clinical Nutrition in Oncology” (7, 14). Studies have found high reliability and validity compared with other screening tools for both outpatients and inpatients and predictive validity to length of hospital stay and mortality rates (16, 17). The MUST is a five step screening tool and takes into account the three variables “actual condition,” “history” and “severity of the current disease” as risk factors. Therefore, the following parameters are required: the patient’s current BMI, the amount of unplanned weight loss within the last three to six months and food abstinence for more than 5 days (steps 1–3). Due to the lack of a definition of food abstinence by the BAPEN, the DGEM definition was chosen. This defines food abstinence as an oral nutritional intake of less than 500 kcal/day (7). For patients in whom height or weight cannot be measured by standard methods or otherwise reliably obtained, alternative procedures, such as estimating the height from measured ulnar length or knee height or estimating BMI from measured mid upper arm circumference (MUAC), are described in detail (15). In the fourth step the overall risk of malnutrition is calculated by adding all scores from step 1–3. Step 5 provides recommendations for action depending on the overall risk identified in all steps (Supplementary material). Moreover, the individual interest in nutrition was determined by a separate patient questionnaire. The first section of the patient questionnaire included three general questions about information needs in the context of nutrition. Patients were initially asked to indicate whether the topic of nutrition in cancer was important to them. In a second question, patients should indicate whether they had a discussion with the treating physician on their individual nutrition. In the third question, patients were asked how they obtain information on the topic of nutrition (such as from physicians, internet, nutritional advice, books, others, with the option to enter free text).

Patients were also asked about dietary changes and weight changes (6 month and 2 weeks ago) as well as adherence to special so called “cancer diets” since their cancer diagnosis. Furthermore, nutritional problems and changes in eating habits occurring during the oncological disease and/or therapy were recorded. Among other things, patients were asked about aversion to certain foods, changes in taste, loss of appetite, and digestive problems. Finally, the type and size of food intake before and after the cancer diagnosis were documented. The patient section of the questionnaire was added as Supplementary material.

### Statistics

The results were statistically analyzed using the data processing program IBM SPSS Statistics (RRID:SCR_016479) version 25 for Windows (18). Besides descriptive statistics analyses, normal distributions were determined using Kolmogorov–Smirnov test and visual inspection of histograms and Q-Q plots. The chi-square test and Kruskal–Wallis test were used to detect correlations and differences. Monte Carlo method was used for expected cell frequencies <5. Effect size was expressed by Phi coefficient (φ) and Cramér’s V and interpreted similarly to a correlation according to Cohen (19). To determine the strength of an association, the effect size r and the odds ratio (OR) were calculated. The significance level was set at *p* < 0.05 for all tests (20).

## Results

### Patient characteristics, anthropometric data and evaluation of the malnutrition universal screening tool

Overall, approximately equal numbers of women and men participated (52 vs. 48%). The mean age was 62.7 ± 11.8 years (Table 1).

**TABLE 1:** Patient characteristic, oncological data and evaluation of MUST-screening.

		♀ 163 (52%)	♂ 148 (48%)	*n* = 311
**Patient characteristic and anthropometric data**				

Age	18–39 years	11 (7%)	5 (3%)	16 (5%)
	40–65 years	94 (58%)	71 (48%)	165 (53%)
	=65 years	58 (36%)	72 (49%)	130 (42%)
BMI	<18.5 kg/m^2^;	2 (1%)	1 (1%)	3 (1%)
	18.5–24.9 kg/m^2^	70 (43%)	58 (39%)	128 (41%)
	25–29.9 kg/m^2^	61 (37%)	53 (36%)	114 (37%)
	=30 kg/m^2^	30 (18%)	36 (24%)	66 (21%)
Unintentional weight loss		73 (45%)	64 (43%)	137 (44%)

**Oncological data**				

Tumor entity	Gynecological	85 (52%)	–	85 (27%)
	Urological	7 (4%)	30 (20%)	37 (12%)
	Gastroenterological	32 (20%)	51 (34%)	83 (27%)
	Hematopoietic	33 (20%)	45 (30%)	78 (25%)
	Dermatological	3 (2%)	13 (9%)	16 (5%)
	Others	3 (2%)	9 (6%)	12 (4%)
Antitumor therapy (currently or within the last six months)	Chemotherapy	131 (80%)	105 (71%)	236 (76%)
	Radiotherapy	8 (5%)	18 (12%)	26 (8%)
	Surgery	48 (29%)	28 (19%)	76 (24%)
Intention to treat	Palliative	115 (71%)	112 (76%)	227 (73%)
	Curative	48 (29%)	36 (24%)	84 (27%)

**Evaluation of the MUST-Screening**				

MUST-score	0	100 (61%)	97 (66%)	197 (63%)
	1	29 (18%)	22 (15%)	51 (16%)
	≥2	34 (21%)	29 (20%)	63 (20%)

MUST, malnutrition universal screening tool; BMI, body mass index; *n*, number (rounding differences are not compensated when totals are calculated).

Evaluating the MUST, in total 20% (*n* = 63) of patients were at high risk of malnutrition, 16% (*n* = 51) were at moderate risk and 63% (*n* = 197) were at low risk (Table 1). Most points were gained by unintentional weight loss. Scoring by BMI or acute disease event occurred in only 5% of patients. In total, half the patients experienced unintentional weight change, with 44% suffering from a weight loss in the six months before survey (M = 9.66 ± 7.27 kg). Regarding this weight loss 65% (*n* = 202) of our cohort reported less than or equal to 5% loss of their body weight. Only one patient gained points for an acute illness event with food abstinence for more than five days.

### Influence of tumor entity, age, therapy and intention to treat on malnutrition risk

**FIGURE 1:**
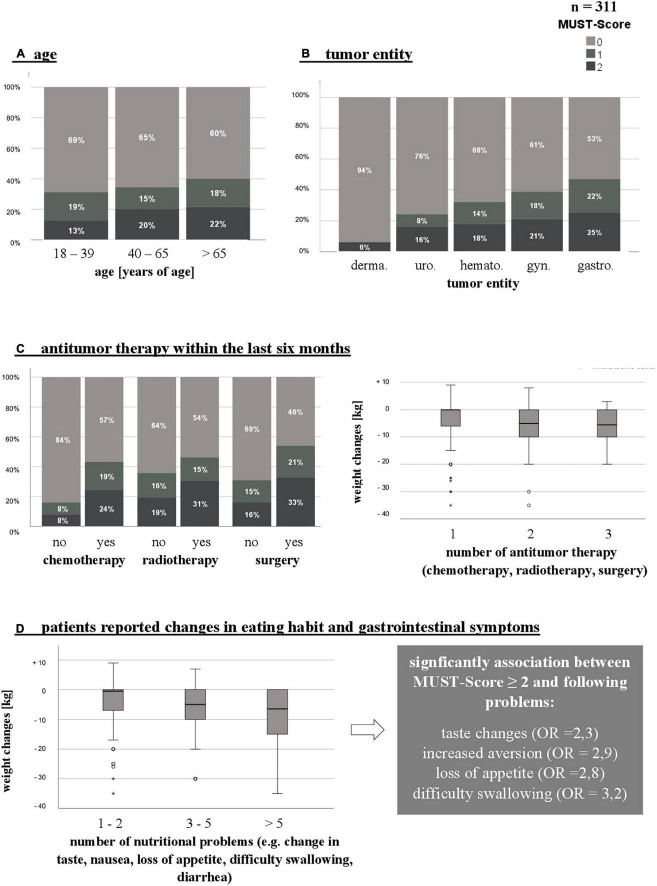
Risk factors that may increase the occurrence of malnutrition (increased risk defined by MUST-Score_2 or unintentional weight loss). **(A)** Age. **(B)** Tumor entity. **(C)** Antitumor therapy within the last 6 months. **(D)** Patients reported changes in eating habits. *n*, number; MUST, Malnutrition Universal Screening Tool; °/*, mild/extreme outliers; OR, odds ratio; derma., dermatological; uro., urological; hemato., hematopoietic; gyn., gynecological; gastro., gastroenterological.

Approximately one quarter of included patients had gynecological, gastrointestinal or hematological malignancies respectively (Table 1). Looking at the mean ranks as well as the graphical representation of the weight changes of the patients surveyed, those with gastrointestinal cancers suffered the highest weight loss and the highest MUST score ≥2 in 25% of patients (Figure 1B; Table 1).

In addition, only a third (33.3%) of patients ≤ 39 years of age lost more than 5 kg body weight, compared to twice as many over 65 years of age (MUST score ≥2 in 22%). However, a significant correlation between unintentional weight loss and age could not be proven (Figure 1A and Table 1). According to gender, increased risk of malnutrition by MUST was about the same in men (*n* = 34, 20%) and women (*n* = 29, 21%).

More than two-thirds (*n* = 215, 69%) of our patient collective received ongoing chemotherapy. Radiation therapy had been performed in approximately 30% (*n* = 93) of patients, while 65% (*n* = 202) has undergone previous surgery for their cancer. Regarding patients’ weight loss, a significant correlation with the timing of surgical tumor therapy as well as chemotherapy could be demonstrated (Figure 1C). Patients receiving surgical treatment in the last six months lost most weight within this period [*X*2 (6) = 20.79; *p* = 0.002, *V* = 0.20]. Similarly, patients receiving chemotherapy at the time or up to 6 months before the survey were particularly affected by unintentional weight loss [*X*2 (8) = 19.42; *p* = 0.013, *V* = 0.24]. In these patients the risk of a MUST score ≥ 2 was three times higher (OR = 3.6). The combination of multiple antitumor therapies had a negative impact on patients’ weight development [*X*2 (6) = 28.53; *p* < 0.001, *V* = 0.30] (Figure 1C).

Regarding treatment 73% (*n* = 227) of patients were in palliative and 27% (*n* = 84) in curative therapy with a prospect of cure. A significant correlation with unintentional weight loss as well as a MUST score ≥ 2 could not be demonstrated.

### Nutritional problems and changes in eating habits

In 57% (*n* = 177) of the patients, relevant nutritional problems (e.g., change in taste, increased sensation of aversion, odynophagia) occurred and changed patients’ dietary behavior and eating habits (Figure 1D). In addition more than half the patients (*n* = 174, 56%) reported problems affecting the gastrointestinal tract. The relation between these factors and weight loss in patients was shown to be highly significant [*X*2 (2) = 40.53; *p* < 0.001, *V* = 0.36]. Almost two-thirds (*n* = 113, 64%) of patients reported that these changes were related to systemic chemotherapy. In 10% (*n* = 17) they occurred together with radiotherapy or other antitumor therapy (e.g., antibody therapy).

The occurrence of taste changes, increased aversion, loss of appetite, and dysphagia can be significantly associated with a MUST score ≥ 2. The risk of developing a poor nutritional status was 2-3-fold higher when one of these problems occurred. Almost a quarter of patients experienced three to five nutritional and gastrointestinal problems and the risk of poor nutritional status increased with the number of problems [*X*2 (6) = 34.86; *p* < 0.001, *V* = 0.34]. Only 25% (*n* = 78) of patients reported none of the above-mentioned issues.

Women were found to be more frequently affected by these problems [*X*2 (1) = 6.61; *p* = 0.01, φ = 0.15]. Dermatological patients seldom suffered from nutritional problems [*X*2 (5) = 21.18; *p* = 0.01, *V* = 0.26]. No significant correlation to nutritional and gastrointestinal problems was found for age, BMI, or treatment.

Furthermore, more than a quarter of patients whose portion size did not change notably nevertheless suffered a weight loss of >5% in the last six months.

Almost all patients, 95% (*n* = 295), could eat solid foods at the time of survey. About 3% (*n* = 9) received additional calories via special high-calorie sip feeding or a PEG tube. Less than 1% of the patients were able to take liquid food only (*n* = 2) or were completely tube-fed (*n* = 1).

### General patient interest in nutrition in cancer and preventive changes in diet

**FIGURE 2:**
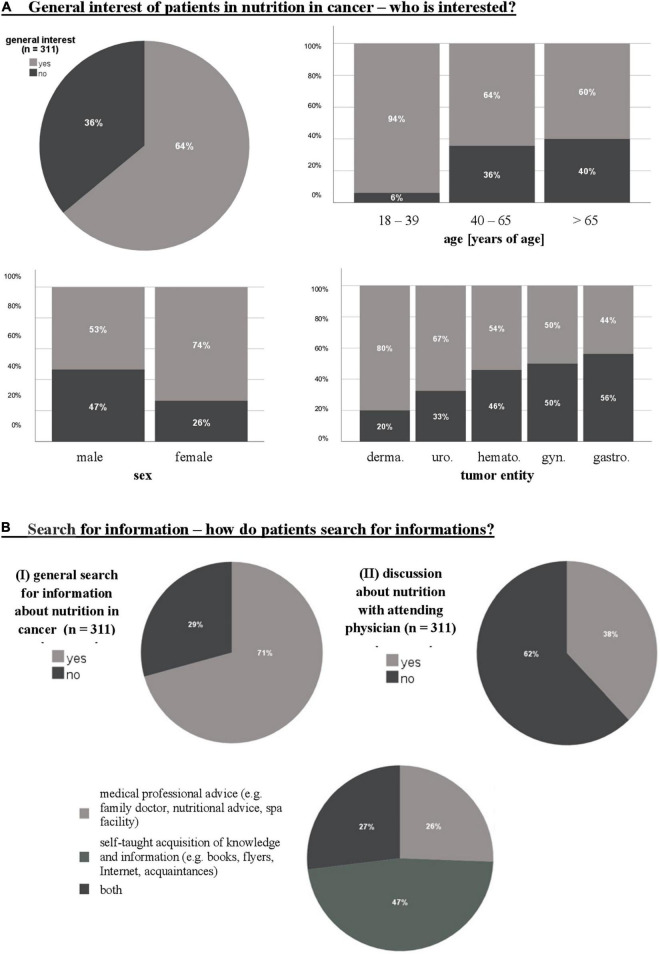
General interest of patients in nutrition in cancer **(A)** and search for information **(B)**. *n*, number; derma., dermatological; uro., urological; hemato., hematopoietic; gyn., gynecological; gastro., gastroenterological.

Sixty-four percent (*n* = 199) of patients showed general interest in the topic of nutrition in cancer (Figure 2A). Overall, a significantly greater interest was found in women [74 versus 53%; *X*2 (1) = 13.79; *p* < 0.001, φ = 0.21]. In addition, interest decreased with increasing patient age with the highest interest in patients up to 39 years of age [*X*2 (2) = 7.05; *p* = 0.029, *V* = 0.15]. Men aged 40 to 65 had the lowest interest. Furthermore, a significant relationship between the type of cancer and the general interest of the patients [*X*2 (5) = 21.58; *p* = 0.001, *V* = 0.26] was evident. Especially patients with gynecological (80%) and gastroenterological tumors (67%) classified nutrition as important. Overweight patients showed less interest than those with normal weight [*X*2 (5) = 16.15; *p* = 0.006, *V* = 0.23]. Furthermore, patients’ interest increased with a higher MUST score and thus with a higher risk of malnutrition [*X*2 (2) = 12.00; *p* = 0.002, *V* = 0.20] independent of the BMI.

The information sources used in this questionnaire can be divided into two groups: 1. medical professional advice (family doctor, nutritional advice, spa facility), and 2. self-taught acquisition of knowledge and information (books, flyers, Internet, acquaintances).

Forty-seven percent of patients acquired knowledge themselves from books and magazines (31%, *n* = 45) with the Internet (22%, *n* = 32) being the most cited source of information. Only a quarter of patients sought information from professional medical providers and only 38% (*n* = 118) of patients had a discussion about nutrition with their attending physician. However, 29% (*n* = 90) of patients also reported no active search for nutritional information (Figure 2B).

In total, 38% (*n* = 118) had changed their diet at the time of cancer diagnosis. With 13% (*n* = 40), only a small proportion of these patients followed a specific “cancer diet,” with low-carbohydrate and ketogenic diets being the most common. However, the majority of patients paid more attention to food selection. For example, 14% (*n* = 44) of patients reported eating a more balanced and healthier diet since their cancer diagnosis. Most patients further specified these statements (e.g., more fruit and vegetables, less meat, less wheat). Women changed their dietary behavior more often than men [62 versus 35%; *X*2 (1) = 4.59; *p* = 0.032, φ = 0.12]. There was also an association between age and dietary change, with 49% of the patients younger than 65 significantly changing their behavior compared to patients older than 65 [23%; *X*2 (2) = 26.15; *p* < 0.001, *V* = 0.29].

## Discussion

In the present study, we could demonstrate that using the MUST, more than one third of oncological outpatients are at risk of developing malnutrition with a high risk for every fifth. This is in line with the results recently published by Hauner et al. for patients in non-university out-patient clinics (12). This prevalence is clearly too high therefore standardized screening procedures and nutritional medical measures should be part of everyday outpatient care. This study confirms that using BMI as a screening parameter and even sole diagnostic criterion must be regarded as critically. Even though an increased MUST score correlates with a lower BMI, only 3 of our patients had a BMI of <18.5 kg/m^2^ and thus should have been classified as malnourished according to the WHO definition. The average BMI was even at 26.5 kg/m^2^, which is overweight. In contrast, according to the MUST score, 63 out of 311 patients (20.3%) are nevertheless at increased risk of malnutrition, with most of the points based on unintentional weight loss (19). Similar results were obtained 2006 in The German Hospital Malnutrition Study by Pirlich et al. (10). Therefore, unintentional weight loss seems to be a more reliable parameter, especially in an increasingly overweight population. Heavy loss of fat-free vital body cell mass and hidden muscle wasting, so called sarcopenic obesity, can be life-threatening regardless of BMI (21). Therefore, multidimensional screening methods recommended by ESPEN and DGEM (e.g., NRS, MUST) should be used at diagnosis, initiation of therapy, and at regular intervals during the course of the disease as recommended in the newly developed Global Leadership Initiative on Malnutrition (GLIM) consensus criteria (7, 22, 23). Furthermore, a higher weighting of dynamic parameters could be useful.

Patients with gastrointestinal tumors suffered the highest weight loss, probably due to more frequent occurrence of nutritional problems. Although age as a risk factor was not highly significant in our analysis, 48% of patients in this subgroup were >65 years old, which could be another risk factor for the development of malnutrition. For example, in the NRS-2002, patients ≥70 receive an additional point. Over all tumor entities we found one in five patients >65 years with a MUST score ≥2. These findings are consistent with former studies, which named type of cancer, advanced age and polypharmacology as key risk factors for malnutrition (10).

In addition to advanced age, we could demonstrate that former or ongoing chemotherapy can be an important risk factor for developing malnutrition. We found a 3.6 fold increased risk of developing malnutrition in patients under and up to 6 months after chemotherapy. Weight loss and poor nutritional status already present in many cancer patients at diagnosis seemed to worsen from chemotherapy side effects. Gastrointestinal symptoms, the most common side effects of chemotherapy, occurred in two-thirds of patients. Furthermore, there was also a significant correlation with tumor surgery in this period (*p* = 0.002). Since the risk of malnutrition increases significantly with increasing therapy, patients with multimodal therapy concepts deserve special attention. As well changes in taste, increased aversion, loss of appetite and dysphagia were associated with a 2-3-fold higher risk of malnutrition. These risk factors were identified as particularly relevant and should therefore be explicitly asked in future screening procedures and counseling sessions. Furthermore, in the present cohort a combination of several nutritional problems leads to a four-time higher risk of malnutrition. This observation is not surprising if one takes into account suspected pathomechanisms of malnutrition in cancer, which makes treatment complex and challenging. Patients with cancer are more likely to be malnourished than patients treated in other specialties. Mechanisms underlying cancer-related impairment of nutritional status include among others indirect effects of cancer and its treatments, metabolic changes in tumor microenvironment and according to systemic inflammation as explained in detail by the “ESPEN expert group recommendations for action against cancer-related malnutrition” (24, 25).

With respect to growing evidence in the research field of gender medicine, the topic of malnutrition should also be further examined on a gender-specific basis. We found women to be significantly more often affected by nutritional problems.

For most patients, especially female and younger ones, it seems also to be important to feel well informed about nutrition. It was striking that overweight patients showed significantly less interest in nutrition. This is also an important finding, because mortality as well as disease progression for some indications is negatively influenced by obesity (26, 27). Obesity is e.g., considered a recognized risk factor for postmenopausal breast cancer (28). Therefore, the current guidelines recommend weight normalization and a healthy nutrition after a cancer therapy, which is key for cancer survivors (29).

Interestingly, patients do not seek information primarily from medical professionals but independently through print media or the Internet. Only one-third consulted their healthcare professionals. Similarly, only 38% of patients had already discussed nutrition with their treating physician.

The results shown here thus correlate with the recently published study by Ostermann et al. Between 2019 and 2020 they surveyed breast cancer patients about their personal experiences with oncological therapy. Sixty-four point five percent of those patients reported not having received any nutritional counseling, although 71.8% of these women would have liked to. In this study 80.8% have taken food supplements without anyone receiving medical consultation on this topic. Due to the lack of nutritional counseling 83.7% informed themselves about therapy-accompanying nutritional options, whereby 71.2% get their information out of the internet. The results of Ostermann et al. and the results shown in our study emphasize the mismatch of the desire for nutritional counseling of patients and the supply reality. Therefore, these studies should encourage physicians to offer nutritional counseling to especially oncological patients (30).

Considering that more than one-third of the monitored patients changed their diet after their cancer diagnosis and 13% followed a specific “cancer diet,” mostly low-carbohydrate or ketogenic, this is an important statement. Due to multifactorial processes, the extent to which conscious changes in diet could have had an influence on the patients’ weight development could not be determined. However, it can be assumed that cancer diets can lead to numerous side effects and even malnutrition (e.g., nausea, poor appetite, weight loss, hypercholesterolemia and pancreatitis) (31). Therefore, the Prevention and Integrative Oncology Working Group of the German Cancer Society advises against a “cancer diet” meaning a low-carbohydrate or ketogenic diet for cancer patients (32). Moreover, to date, the majority of restrictive “cancer diets” have not been shown to be effective in regressing tumors, improving treatment response, reducing side effects, or prolonging survival (33).

### Limitations of the study

As all interviews took place during systemic therapies, patients’ height and weight were asked and not measured on site. Neither was information on weight loss was collected by history and not verified on-site. Therefore, it is possible (especially on the subject of absolute weight) that answers were given in terms of presumed desirability. Since the patients’ questionnaire was usually filled out together with the patients and some questions were rephrased and examples given in case of ambiguities, it is conceivable that this could have influenced certain answers. Nutritional status and habits before tumor diagnosis can be an important variable in malnutrition. Regarding dietary habits, patients were asked to indicate whether they had changed their diet and whether they had changed their portion sizes since disease onset. No other information was obtained in this survey, which retrospectively would have been desirable. Furthermore, it would be valuable to know in more detail what patients are specifically interested in (e.g., nutrition composition), which was not subject of the questionnaire. However, since this information in particular would be an important feedback for the adaptation of the counseling and information offers, oncological patients should be given the opportunity to report back in future surveys which information and training offers would be helpful and relevant.

In addition 42% of the study participants were older than 65 years. For this reason, it would be important to include other risk factors that are potentially relevant for this age group in clinical routine screening (e.g., mobility, neuropsychological problems), as is the case with the Mini Nutritional Assessment (MNA) ^®^ (34).

### Conclusion

Nevertheless, in addition to validated screening procedures, our results demonstrate, that adequate nutritional medical counseling needs to become part of the oncological therapy. A healthy, balanced diet and lifestyle play a crucial role not only in prevention but also in the treatment of cancer.

As to conclude, nutritional medicine is considered an integral part of the therapy of oncological patients. Still, physicians and patients must be made aware of the risks of untreated malnutrition. As our results demonstrate, regular nutritional status recording and systematic continuous screenings for malnutrition should be performed in all oncological patients. It is, however, even more important for outpatients and is especially key during pandemics, as now. In addition to reliable screening instruments like the MUST score, individual risk factors depending on type of cancer, gender and age should be integrated into the nutritional screening process to improve patients’ quality of life, treatment adherence and probably even prognosis. Furthermore, as we could show, nutritional counseling is an important resource in tailored oncology concepts to address patients’ information about healthy eating and to empower their self-awareness of their own dietary behavior, weight history, and related physical condition. This would give patients the opportunity to actively participate in coping with the disease.

## Data availability statement

The original contributions presented in this study are included in the article/Supplementary material, further inquiries can be directed to the corresponding author.

## Ethics statement

The studies involving human participants were reviewed and approved by Ethics Committee of the University of Würzburg. The patients/participants provided their written informed consent to participate in this study.

## Author contributions

LS, CL, and HE contributed to the study concept. JJ, CL, CW, LS, MW, and DH contributed to the methodology. JJ and DH drafted the manuscript. MW, CL, LS, CW, AM, and HE critically reviewed and edited the article. JJ and CL carried out the investigation. JJ contributed to the data curation and visualization of the data. JJ, DH, MW, and CL were responsible for the formal analysis. HE contributed the supervision. All authors have read and agreed to the published version of the manuscript.
