# Corrigendum: Every fifth patient suffered a high nutritional risk – Results of a prospective patient survey in an oncological outpatient center

**DOI:** 10.3389/fnut.2023.1140692

**Published:** 2023-03-23

**Authors:** Julia Jendretzki, Dorothea Henniger, Lisa Schiffmann, Constanze Wolz, Anne Kollikowski, Alexander Meining, Hermann Einsele, Marcela Winkler, Claudia Löffler

**Affiliations:** ^1^Comprehensive Cancer Center, University Hospital Würzburg, Würzburg, Germany; ^2^Klinik Hallerwiese-Cnopf'sche Kinderklinik, Neonatologie und Pädiatrie, Nuremberg, Germany; ^3^Department of Internal Medicine II, University Hospital Würzburg, Würzburg, Germany; ^4^Robert-Bosch-Krankenhaus, Stuttgart, Germany

**Keywords:** nutritional risk screening, malnutrition, nutritional counseling, oncology outpatients, MUST-Score, nutritional medical needs

In the published article, there was a mistake in the **Funding** statement. After publishing the article, the library of the University of Wuerzburg offered an Open Access Publication Fund to the authors. The corrected funding statement appears below:

This publication was supported by the Open Access Publication Fund of the University of Wuerzburg.

The authors apologize for this error and state that this does not change the scientific conclusions of the article in any way. The original article has been updated.

