# Supplementary material for: Every fifth patient suffered a high nutritional risk—Results of a prospective patient survey in an oncological outpatient center
Source: Front Nutr. 2022 Oct 31;9:1033265. doi: 10.3389/fnut.2022.1033265 (PMC9659884; doi:10.3389/fnut.2022.1033265)
Supplement: Supplementary file 1 [file Data_Sheet_1.PDF]

Questionnaire – to be completed by patients with the examiner

Date:

Patient ID: ______________________ ☐ male ☐ female

Dear Patient,

This is a questionnaire on the topic of **nutrition during cancer**. It is part of a research project and is intended to demonstrate how important nutrition is for our oncology patients and how many patients would benefit from nutrition counselling and/or nurtition therapy in addition to traditional medicine.

Introduction:

The questionnaire has two parts (general questions as well as special questions about your personal nutrition and any personal nitrition issues). The data from the questionnaire is compiled and evaluated in compliance with statutory data protection rules and treated strictly confidentially.

1. **General questions:**

I Is the topic „nutrition during cancer" important for you?

☐ Yes ☐ No

II Have you spoken to a doctor about nutrition or been asked about nutrition by a doctor in relation to your illness?

☐ Yes ☐ No

III How do you find information about nutrition, if this is something you do?

☐ doctor ☐ nutrition counselling
☐ Internet ☐ bookshop
☐ other: ______________________

IV Have you changed your diet since the beginning of your illness (e.g. certain „cancer diets“ such as a ketogenic diet, the Budwig diet, low carb etc)?

☐ Yes, I have changed my diet as follows: ______________________________

If you have answered the first question with „yes“, does this refer to your current diet?

☐ Yes ☐ No

____

☐ No, I have not changed my diet.

2. Questions on your nutrition / nutrition problems:

I Details about weight

1. In the past 6 months:

☐ Loss: kg ☐ Gain: kg ☐ unchanged

2. In the past 2 weeks:

☐ Loss: kg ☐ Gain: kg ☐ unchanged

II Have you changed your usual diet?

☐ Yes, since: ________

☐ disgust of, aversion to certain foods, e.g.: ______________

☐ change of taste, e.g.: ______________

☐ pain when eating, e.g.: ______________

☐ other reasons: ______________

☐ No

III What kind of food can you eat?

☐ usual, solid food ☐ pureed light diet

☐ only liquids (clear soups, drinkable food)

☐ low-calorie liquids (tea, water)

☐ no food

IIII Are you suffering from or have you recently suffered from nutrition problems?

☐ Yes, from:

☐ loss of appetite ☐ nausea ☐ vomiting
☐ problems swallowing ☐ diarrhoea ☐ constipation
☐ other complaints: ______________________

☐ No

IV How does the size of your current portions differ from before your illness?

☐ unchanged (100%)

compared to before
☐ approx. three-quarters
☐ approx. half
☐ approx. a quarter

Thank you very much for taking the time to complete this questionnaire. If you feel that you would like to learn more about nutrition or if today's questionnaire has shown that you could perhaps have a nutrition deficiency, we would like to draw your attention to the nutrition counselling service offered by the oncology centre.

Would you like to make an appointment for nutrition counselling?
Please contact us using the flyer for out-patient nutrition counselling or call us direct to arrange an appointment
